# Efficacy of confrontational counselling for smoking cessation in smokers with previously undiagnosed mild to moderate airflow limitation: study protocol of a randomized controlled trial

**DOI:** 10.1186/1471-2458-7-332

**Published:** 2007-11-15

**Authors:** Daniel Kotz, Geertjan Wesseling, Marcus JH Huibers, Onno CP van Schayck

**Affiliations:** 1 Department of General Practice, School for Public Health and Primary Care (CAPHRI), Maastricht University, Maastricht, The Netherlands; 2 Department of Respiratory Medicine, Maastricht University Hospital, Maastricht, The Netherlands; 3 Department of Clinical Psychological Science, Maastricht University, Maastricht, The Netherlands

## Abstract

**Background:**

The use of spirometry for early detection of chronic obstructive pulmonary disease (COPD) is still an issue of debate, particularly because of a lack of convincing evidence that spirometry has an added positive effect on smoking cessation. We hypothesise that early detection of COPD and confrontation with spirometry for smoking cessation may be effective when applying an approach we have termed "confrontational counselling"; a patient-centred approach which involves specific communication skills and elements of cognitive therapy. An important aspect is to confront the smoker with his/her airflow limitation during the counselling sessions. The primary objective of this study is to test the efficacy of confrontational counselling in comparison to regular health education and promotion for smoking cessation delivered by specialized respiratory nurses in current smokers with previously undiagnosed mild to moderate airflow limitation.

**Methods/Design:**

The study design is a randomized controlled trial comparing confrontational counselling delivered by a respiratory nurse combined with nortriptyline for smoking cessation (experimental group), health education and promotion delivered by a respiratory nurse combined with nortriptyline for smoking cessation (control group 1), and "care as usual" delivered by the GP (control group 2). Early detection of smokers with mild to moderate airflow limitation is achieved by means of a telephone interview in combination with spirometry. Due to a comparable baseline risk of airflow limitation and motivation to quit smoking, and because of the standardization of number, duration, and scheduling of counselling sessions between the experimental group and control group 1, the study enables to assess the "net" effect of confrontational counselling. The study has been ethically approved and registered.

**Discussion:**

Ethical as well as methodological considerations of the study are discussed in this protocol. A significant and relevant effect of confrontational counselling would provide an argument in favour of early detection of current smokers with airflow limitation. Successful treatment of tobacco dependence in respiratory patients requires repeated intensive interventions. The results of this study may also show that respiratory nurses are able to deliver this treatment and that intensive smoking cessation counselling is more feasible.

**Trial registration:**

Netherlands Trial Register (ISRCTN 64481813).

## Background

Chronic obstructive pulmonary disease (COPD) is a preventable and treatable disease which is characterized by airflow limitation that is not fully reversible [1]. Spirometry is the gold standard for the diagnosis and assessment of the disease [1]. COPD is currently the fifth leading cause of death worldwide [2], and projections for 2020 indicate further increase in global mortality, placing COPD on the third position of lethal diseases [3]. Cigarette smoking is by far the most important risk factor for COPD, and smoking cessation is the single most effective way to reduce the risk of developing COPD and to affect the outcome in patients at all stages of the disease [4,5].

Underdiagnosis of COPD is a worldwide problem [6]. Most patients present to their doctor for various other reasons but often have respiratory symptoms, and in those who do present with respiratory symptoms, COPD is not always suspected nor diagnosed [7]. Because of the irreversible and progressive nature of the disease, early intervention is important. However, the use of spirometry for early detection of COPD is still an issue of debate [8-10]. The most important counterargument is that there is no convincing evidence that spirometry has an added positive effect on smoking cessation [11-13].

### Why use spirometry for smoking cessation?

In theory, spirometry might be useful as a motivational tool for smoking cessation in smokers who are at risk of developing (or have) COPD. While most smokers acknowledge that smoking is dangerous, many trivialize their own perceived risk of the disease, or deny or avoid information about the dangers of smoking in order to reduce cognitive dissonance [14-18]. One might therefore expect that confronting smokers with an objectively (by spirometry) identified negative consequence of smoking (airflow limitation) positively affects the outcome of their quit attempt. This idea was already proposed in the 1960's by Peters and Ferris who argued that assessing the negative effects of smoking on lung function "might serve as a lever to influence the young adult to reduce his smoking habits" [19]. Since then, various studies have been performed to study the efficacy of spirometry for smoking cessation. The results, however, are inconclusive as shown in a the systematic review by Wilt et al. on spirometry as a motivational tool for smoking cessation [11,12]. Also, previous studies have one or more important methodological limitations such as unstandardized counselling intensity, incomparable or uncontrolled use of pharmacological aids for smoking cessation between experimental and control group, and different (or unclear) baseline levels of lung function and motivation to quit smoking. More well-designed research is needed to assess the efficacy of spirometry for smoking cessation.

### Hypothesis and research questions

We hypothesise that early detection of COPD and confrontation with spirometry for smoking cessation may be effective if the following approach we have termed "confrontational counselling" is applied [20]. Confronting patients with COPD is not an isolated approach but should be integrated into state-of-the-art smoking cessation treatment. Confrontational counselling should consist of several counselling sessions on an individual, face-to-face level, under supervision of a trained smoking cessation specialist, and in combination with evidence-based pharmacological treatment for smoking cessation.

Our primary research question is: what is the efficacy of confrontational counselling in comparison to regular health education and promotion for smoking cessation delivered by specialized respiratory nurses in current smokers with previously undiagnosed mild to moderate airflow limitation (i.e. GOLD [1] stage 1 and 2 COPD) with regard to prolonged abstinence from smoking during a period of 12 months?

In this group of smokers with previously undiagnosed mild to moderate airflow limitation we want to address the following secondary research questions:

1. Which baseline characteristics are predictors of outcome (i.e. 12-month prolonged abstinence from smoking)?

2. What are the effects of early detection of airflow limitation and smoking cessation on lung function, perceived specific health-related complaints, quality of life, and mental health after 12 months follow-up?

3. What is the cost-effectiveness and cost-utility of early detection in combination with confrontational counselling delivered by respiratory nurses?

4. What are the effects of labelling of disease (COPD) on self-efficacy, perceived health status, quality of life and mental health?

5. What are the ethical considerations of early detection of airflow limitation and subsequent confrontational counselling for smoking cessation?

## Methods/Design

### Study design

**Figure 1:**
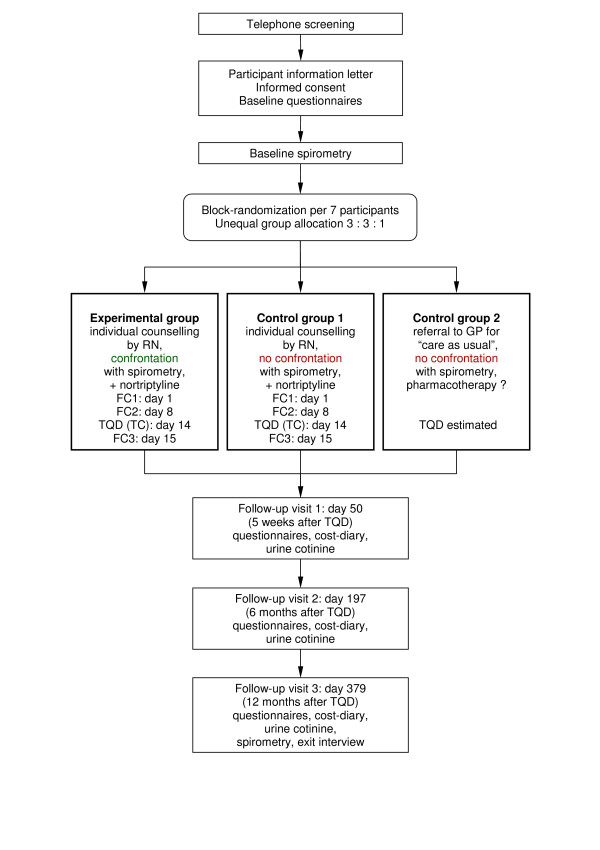
**Design**. FC = face-to-face counselling session; TC = telephone counselling session; TQD = target quit date; RN = respiratory nurse; GP = general practitioner.

In short, the design of this study is a randomized controlled trial comparing confrontational counselling delivered by a respiratory nurse (RN) combined with nortriptyline for smoking cessation (experimental group), health education and promotion delivered by a RN combined with nortriptyline for smoking cessation (control group 1), and "care as usual" delivered by the GP (control group 2). Early detection of smokers with mild to moderate airflow limitation is achieved by means of a telephone interview in combination with spirometry. For an overview of the study design see figure 1.

The efficacy of smoking cessation interventions in clinical studies depends on the characteristics of the study population, the intensity of behavioural support, and the use of pharmacological aids for smoking cessation [21]. In order to isolate the effect of confrontational counselling on smoking cessation, other factors that are associated with the outcome must be standardized between the comparison groups. All participants of this trial have previously undetected mild to moderate airflow limitation. Participants from both the experimental group and control group 1 receive an equally intensive counselling (in terms of number, duration, and scheduling of counselling visits) and dosage of nortriptyline for smoking cessation. Another reason for using an active control is that it would not be ethically sound to withhold smokers with airflow limitation from smoking cessation treatment.

### Sample size calculation

The primary research question aims at a contrast in efficacy between the experimental group (confrontational counselling) and control group 1 (health education and promotion). Therefore, the calculation of the sample size is based on the identification of a difference in proportion of prolonged abstinence after 12 months between these two groups. We estimated the relevant difference in proportion to be 15%: 35% quitters in the experimental group versus 20% quitters in control group 1. When putting the risk of a type I-error at 5% and the risk of a type II-error at 20%, 136 participants per group are needed at onset to detect a difference in proportions of 15%. Considering 10% lost to follow-up, 150 participants per group are needed in the experimental group and control group 1 (136 × 0.9^-1^). We expect a larger difference between the experimental group (35%) and control group 2 (8% [22]). Therefore, less participants are needed in control group 2. We used a formula for the calculation of sample sizes of unequal groups and set the ratio between the experimental group and control group 2 at 3:1. This resulted in a minimum of 32 participants in control group 2 (taking into account 10% lost to follow-up).

### Preparation of the trial

In preparation of the trial, all GPs in Dutch and Belgian Limburg (the area surrounding the city of Maastricht) have been informed about the study. We have prepared an office for the screening, counselling, and follow-up visits of participants at Medical Centre Annadal, Maastricht. We have built a relational database for the control of all study events and the collection of data. This is very important because of the complexity of the study.

### Recruitment of participants

Subjects are recruited in the general population (through advertisements in local newspapers, flyers, posters, and mailings to households) and in primary care practices (during consultations and through posters and personalized mailings) in Dutch and Belgian Limburg. The text in the advertisements, on flyers, and on posters explains that Maastricht University is performing a study on smoking cessation treatment in which individual counselling is combined with medication for smoking cessation. Current smokers aged 35 to 70 years, who are motivated to quit smoking, are asked to contact us by telephone or by e-mail. We also refer to a website with information about this study. No information about the target condition we are looking for (airflow limitation) is given to participants during recruitment.

### In- and exclusion criteria and process of eligibility screening

Eligibility is screened in two steps; during an initial telephone interview followed by spirometry. The following inclusion criteria are checked during the telephone interview: smoking history of 10 or more pack years; being motivated to stop smoking; being competent to read and speak Dutch; and reporting a respiratory symptom, defined as an affirmative answer to at least one of the following three questions: "Do you cough regularly?", "Do you cough up phlegm (sputum) when you don't have a cold?" or "Have you been shorter of breath lately?". Exclusion criteria are: evidence of a prior respiratory diagnosis, defined by an affirmative answer to the question "Do you have COPD, chronic bronchitis, asthma or asthmatic bronchitis?". Participants are also not allowed to have undergone a lung function test (spirometry) during the preceding 12 months. One or more contraindications for using the smoking cessation medication (nortriptyline) are also reasons for exclusion, among others the current use of anti-depressants. Nortriptyline is a tricyclic anti-depressant which should not be used for smoking cessation in conjunction with another anti-depressant. After the end of the telephone interview, an appointment for spirometry at Medical Centre Annadal is scheduled. Subjects are eligible for participation who have airflow limitation defined as post-bd. FEV_1_/FVC < 70% in combination with post-bd. FEV_1_≥ 50% of predicted value; i.e. mild (GOLD 1) or moderate (GOLD 2) airflow limitation, according to the international GOLD guideline [1]. The results of spirometry are not discussed during or directly after spirometry.

Subjects with severe airflow limitation (post-bd. FEV_1_/FVC < 70% in combination with post-bd. FEV_1 _< 50% of predicted value) are excluded from participation and advised to contact their GP or a lung physician for further evaluation. Subjects without airflow obstruction (post-bd. FEV_1_/FVC ≥ 70%) are also excluded. These smokers are told that despite their normal lung function, they still are at risk of getting other smoking related diseases which are not measured by spirometry, such as cancer or cardiovascular disease. They are strongly recommended to give up smoking. Both groups of excluded smokers get the advice to stop smoking and receive a box with information material about all existing therapies for smoking cessation from the Dutch foundation for a smoke free future (STIVORO).

### Spirometry

Spirometry is performed by two qualified research assistants under permanent supervision of a pulmonologist (GW) according to the criteria of the American Thoracic Society (ATS)/European Respiratory Society (ERS) task force for standardization of lung function testing [23,24] using a Vitalograph^® ^2120 (Vitalograph Ltd, Buckingham, England). After a minimum of three acceptable and reproducible FVC manoeuvres, a bronchodilator (500 μg terbutaline) is administered to the subject in preparation for the reversibility test. After 15 minutes, another series of three FVC manoeuvres is performed. All spirometric test results are independently validated by a pulmonologist (GW) and by a specialised lung function laboratory assistant who was not involved in the trial. In case of initial disagreement, consensus is obtained during re-examination.

### Informed consent procedure

Written information about the study is sent to the candidate participant after the telephone interview, along with the informed consent form. The candidate has at least one week time for reflection before spirometry and can contact the researcher or the research assistant (RA) for further information at any time. The informed consent form is signed by the participant in presence of the RA during the visit for baseline spirometry.

The participant information letter gives information about the existence of two intervention groups only. It says that in one group participants receive "care as usual" by their own GP (this is control group 2) and in the other group participants receive treatment from a trained RN. The latter group is in fact a combination of the experimental group (confrontational counselling by RN + nortriptyline) and control group 1 (health education and promotion by RN + nortriptyline). These two groups are identical with regard to the number and duration of counselling sessions and the use of nortriptyline, but differ only concerning the content and style of the behavioural support: confrontational counselling versus health education and promotion. Just this difference in content must not explicitly be mentioned in the participant information letter in order to safeguard the internal validity of the study. We would jeopardize the idea behind early detection of patients with airflow obstruction by means of spirometry if we would speak about "confrontational counselling" or mention the target condition (airflow limitation). Participants must not know that we use results from spirometry as part of one intervention. The design we use is adapted from Zelen's design [25,26] which may be particularly useful when evaluating the full unbiased impact of screening interventions [27].

At the end of the study, after the 12-month follow-up visit, all participants will indeed be fully informed about the real nature of the study. All participants and their corresponding GPs will be informed about the result of the spirometry. If a GP needs the results of spirometry for the regular care of his/her patient before the end of the study, the required information will be provided. This procedure is approved by the medical ethics committee of Maastricht University and Maastricht University Hospital.

### Randomization and planning procedure

All eligible subjects with previously undetected mild to moderate airflow limitation are contacted by telephone a few days after baseline spirometry to be randomised to one of the three intervention groups (apart from those candidates who changed their mind and who are no longer willing to participate in the study). Also at this moment, the results of spirometry are not discussed. The database of the trial incorporates a randomization system of seven participants per block, allowing an unequal group allocation of 3 : 3 : 1; experimental group : control group 1 : control group 2. When eligible subjects are contacted by telephone, the RA randomises the subject by pressing a button on the computer screen. The database then randomly allocates the subject. Neither the primary researcher nor any other person involved in the study can predict or influence which treatment group the next participant will be allocated to.

After randomization, all treatment and follow-up visits are planned for the whole study period. A schedule with all visits is sent to the participant. At the same time, the GP of each participant is sent a letter informing the GP that the participant is taking part in the study.

### Experimental group and control group 1: L-MIS as the common basis of counselling

Participants from both the experimental group and control group 1 receive counselling delivered by a RN combined with nortriptyline for smoking cessation. The common basis for the counselling is the so-called "L-MIS" protocol for the treatment of nicotine and tobacco addiction which has been implemented among all RNs in the Netherlands in recent years [28].

The number of counselling sessions, their duration and scheduling is fixed in both the experimental group and control group 1 (see also figure 1). The first face-to-face counselling session (FC1; day 1, duration 40 minutes) starts with getting acquainted with each other. The RN tries to build up a relationship with the participant which is based on trust. The participant's smoking characteristics are defined by asking about smoking status, cigarettes smoked per day, and readiness to quit. Nicotine addiction is assessed by number of cigarettes per day and moment of smoking the first cigarette after waking up in the morning. The motivation for quitting smoking is assessed and increased by asking the readiness for quitting and reasons for smoking and quitting. Also, the health risks of smoking are discussed and the pros of quitting. The use of nortriptyline for smoking cessation is discussed, including mechanism, dosage, administration, and possible side effects (for more information about the study medication see paragraph "use of nortriptyline for smoking cessation"). The participant starts with the intake of the study medication the same day.

At the beginning of the second face-to-face counselling session (FC2; day 8, duration 40 minutes), the use of the study medication is evaluated and possible side effects are discussed. Barriers of quitting and the most important problems with previous quit attempts are discussed. The RN tries to increase the participant's self-efficacy towards smoking cessation. The focus of the second session is to prepare the participant for the target quite day (TQD). The RN discusses with the participant how to deal with the most important barriers of quitting smoking. The RN anticipates problems with withdrawal, difficult moments, and craving. The RN provides pointers for the TQD and schedules the telephone counselling on that day.

On the TQD (day 14), the participant is counselled on the telephone (TC; duration 5 – 15 minutes). The RN evaluates the quit attempt, discusses difficult moments, and gives advice for quitting and abstaining from smoking.

The third face-to-face counselling session is scheduled directly after the TQD (FC3; day 15, duration 40 minutes) and starts with an evaluation of the quit attempt and the use of the study medication (including possible side effects). The RN discusses with the participants what is going well and what is problematic with this quit attempt. The RN identifies difficult moments and strategies to deal with theses situations in the future. Participants who did not quit smoking yet or who already relapsed are asked about their reasons and are encouraged to try again.

The fourth face-to-face counselling session (FC4; day 22, duration 40 minutes) basically resembles the third session; the RN evaluates the quit attempt and the use of the study medication. As this is the last counselling session, the focus lies on preparing the participant for continuation of the quit attempt during the follow-up period. The participant is asked to continue with the intake of nortriptyline according to the protocol. At the end of the session, the RN asks the participant's feedback on the perceived effectiveness of the behavioural support and the study medication.

All RNs have had initial training in the use of the L-MIS method and are experienced in the treatment of nicotine addiction. All RNs are trained to use a tailored version of the L-MIS protocol which is specifically designed for this trial. The compliance of RNs with the treatment of participants is stimulated by the use of a protocolized treatment manual, including intervention registration forms providing information per session about all the aspects of smoking cessation counselling to be addressed. The RNs are trained to fill out these intervention forms during each counselling session.

### Discriminative component in the experimental group: confrontational counselling

The number of counselling sessions, the duration, and the scheduling are identical in the experimental group and control group 1. However, specific aspects of "confrontational counselling" are added to the L-MIS in the experimental group which discriminate the treatment from the treatment in control group 1. These aspects derive from the principles of "confrontational counselling" which we have described in more detail elsewhere [29]. Confronting patients with COPD is not an isolated approach but should be integrated into state-of-the-art smoking cessation treatment. Confrontational counselling should consist of several counselling sessions on an individual, face-to-face level, under supervision of a trained smoking cessation specialist, and in combination with evidence-based pharmacological treatment for smoking cessation.

**Figure 2:**
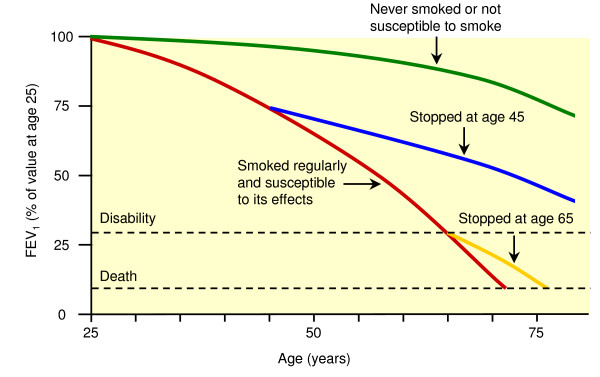
**"Fletcher curve"**. Adapted from Fletcher & Peto (1977): The natural history of chronic airflow obstruction [30].

A key element of confrontational counselling is to confront the participant with his/her airflow limitation during the first counselling session (FC1). The RN discusses the results of the participant's individual baseline spirometry and explains the manifestations of COPD; COPD is a slowly progressive, irreversible but treatable disease. Most importantly in this respect is to make the participant understand that his/her cigarette smoking is the primary cause of the disease. The negative effect of smoking on the lungs is illustrated by showing and comparing images of a normal lung and a "smoker's lung". The participant is asked to recognize common symptoms, functional limitations, and participation problems associated with COPD. The natural history of COPD is discussed and illustrated using the so-called "Fletcher curve" (see also figure 2) [30]. Confronting patients with a serious disease that has a bad prognosis arouses fear. Fear arousal is not a goal in itself and it is not likely to automatically lead to the desired action (smoking cessation). Therefore, when discussing the results of spirometry, the RN tries to make the participant understand that there is an effective and feasible therapy for the disease: smoking cessation. Because airflow limitation has been detected early in the participant, early treatment is possible to avoid further damage. Smoking cessation is the only therapy to reduce the progression of the disease resulting in prolonged life expectancy and improved quality of life. The motivation of the participant to stop smoking in combination with the behavioural counselling offered by the RN and the smoking cessation medication (nortriptyline) increases the chance of the participant to quit smoking and to subsequently improve health. At the end of the session, the participant receives a folder with background information on COPD which is developed by the Dutch College of General Practitioners (NHG). In contrast to the experimental group, participants from control group 1 are not being confronted with the detected airflow limitation. The RN from control group 1 is instructed not to discuss the result of spirometry at any time, but to treat the participant as a "healthy smoker".

The information about airflow limitation and COPD during the first counselling session probably has impact on the participant. At the beginning of the second counselling session (FC2), the RN asks the participant to reflect on this information. The RN assesses whether the participant has processed the information correctly and provides feedback on the thoughts, feelings, and beliefs the participant reports. Again, the positive effect of smoking cessation on the history of lung function is stressed and illustrated using the Fletcher curve. This is repeated during later counselling sessions if necessary.

Confrontational counselling comprises more than merely confronting the participant with his/her results from spirometry. It is a patient-centred approach which involves specific communication skills and elements of cognitive therapy. An important condition is a relationship (also known as alliance) between RN and participant in which both roles are equivalent (rather than an expert-recipient relationship). The RN respects the participant's freedom of choice regarding his/her own smoking behaviour. The RN stimulates the participant to reflect on his/her smoking behaviour by carefully listening to the participant's story, using open ended questions, paraphrases, and reflections. Confrontational counselling aims to identify certain cognitions about smoking such as health concerns, risk perception and self-exempting beliefs. The RN tries to challenge irrational beliefs about smoking by raising the smokers consciousness about these beliefs, testing their reality, and by exploring the relationship between beliefs and behaviour. An example for a typical self-exempting belief of a smoker would be: "Smoking is possibly not very harmful because many smokers live long. My grandfather is 85 and he smoked all his life". The RN will try to challenge this belief for instance by conduction an objective risk assessment, or by exploring biases in the belief itself.

At the end of the first, second, and third counselling session, the RN hands out a smoking cessation diary to the participant. Homework is an essential element in cognitive therapy, and self-monitoring diaries are used as extra input for the counselling sessions. In the first diary (evaluated during FC2), participants have to record their smoking behaviour; they have to count the number of cigarettes smoked and have to describe one situation in which they experience great desire to smoke. In the second diary, participants have to record their thoughts when smoking. Again they have to count the number of cigarettes and have to describe one situation, but also what was on their mind directly before and after lighting up. In the third diary (evaluated during FC3), participants have to describe both behaviour and thoughts in situation when they have great desire to smoke.

To ensure the building of an alliance between RN and participant, it is important the participant is counselled by one and the same pulmonary nurse during the whole intervention period to prevent contamination between the groups. Consequently, each treatment group has its own RNs. All RNs have had initial training in the use of the L-MIS method and are experienced in the treatment of nicotine addiction. Additionally, RNs from the intervention group will receive a four-hour group training in confrontational counselling. The group training is lead by a cognitive therapist (MJH) who acts as supervisor throughout the study, and incorporates practical training with a simulation patient. Supervision meetings between RNs and the supervisor will be planned every 6 weeks during the whole intervention period of the trial.

RNs from control group 1 do not receive an introductory group training but receive feedback during regular evaluation meetings between the RN and the principal investigator.

### Control group 2: care as usual by GP

Participants from control group 2 are referred to their own GP for smoking cessation treatment. They are asked to make an appointment with their GP within the next ten days. They are provided with a referral letter explaining to the GP that the person is participating in a study on smoking cessation. This letter does not give any information about the results from spirometry and the fact that the participant has airflow limitation. The GP is asked to provide the care he/she usually provides to patients who want to quit smoking. In the Netherlands, primary "care as usual" for smoking cessation involves the use of a protocol for low intensity health education and promotion, the so-called "H-MIS" [22]. According to the protocol of the H-MIS, the GP and/or the assistant takes the following steps to assist the smoker during a quit attempt: determine the smoking profile of the smoker, determine the motivation to stop smoking (and increase the motivation if necessary), talk about the barriers of quitting smoking, set a target quit date, discuss the use of smoking cessation aids, and arrange follow-up. A semi-structured interview will be used among participants from control group 2 during the first follow-up visit (day 50) in order to assess whether participants have consulted their GP and which treatment for smoking cessation the GP has delivered.

Neither participants from control group 2 nor their GP will be informed about the results from spirometry and the detected airflow limitation. However, if a GP explicitly requests the results from spirometry they will be provided (see further paragraph "ethical considerations").

### Use of nortriptyline for smoking cessation

Previous research has shown that the combination of counselling and pharmacotherapy is more effective than either alone [31-33] and international guidelines recommend the use of pharmacotherapy in all patients trying to make a quit attempt [21,34]. Participants from both the experimental group and control group 1 receive an equal dosage of nortriptyline (Nortrilen^®^) for smoking cessation. Nortriptyline is a tricyclic anti-depressant which has been shown to be a cheap and effective alternative for the anti-depressant bupropion (Zyban™) [31,35]. Participants start taking nortriptyline on the day of the first counselling visit (FC1, day 1) in which they receive instructions about the use of nortriptyline by the RN. A run-in period of 10 days until the TQD is needed to achieve steady-state blood levels of nortriptyline. From day 1 through day 3, participants take one pill of 25 mg nortriptyline once a day (preferably after dinner). From day 4 through day 7, participants take 50 mg a day (given as two pills of 25 mg). As from day 8 through the end of the treatment period (day 49), participants take 75 mg a day (given as three pills of 25 mg). The RN monitors the correct use of the medication and the occurrence of side-effects during the intervention period. In case of unpleasant or severe side-effects, the dosage will be reduced or the use of the medication will be stopped. At the first follow-up visit (day 50) the RA collects and counts the remaining pills.

### Follow-up visits

Three follow-up visits for all participants are scheduled at day 50 (approximately five weeks after the TQD), day 197 (approximately six months after the TQD), and day 379 (approximately twelve months after the TQD; see also figure 1). The TQD in participants from control group 2 is set at 8 weeks after the day participants were randomized and informed about group allocation. We estimated that this would be sufficient time to schedule a consultation with the GP and to prepare stopping smoking, and that the time lag between day of randomization and TQD would be about the same compared to the experimental group and control group 1 (in the latter groups, we account for a delay between day of randomization and start of treatment). Participants receive a reminder letter including a follow-up questionnaire and a cost-diary seven weeks prior to all three visits. The RA calls every participant one week prior to the visit to confirm the appointment. Minor deviations from the scheduling of follow-up visits will be allowed in order to retain as many participants in the study as possible.

At every follow-up visit, participants hand in their questionnaire and cost-diary. The RA briefly discusses the quit attempt. Urine is collected from every self-reported non-smoker for the analysis of cotinine levels.

During the final follow-up visit, spirometry is repeated in all participants. All spirometric outputs are carefully evaluated by a pulmonologist (GW) and reported to the participant's GP by letter. All participants are asked to consult their GP for information about their lung function and further treatment.

### Data collection

An overview of all measurements per visit is given in table 1. The paper-and-pencil questionnaires are filled out at home by the participants and are handed in during the visits. Completion of the questionnaires takes about 30 minutes. Data from the questionnaires will be double-entered and checked by blinded assistants from the centre for data and information management of Maastricht University (MEMIC).

**Table 1:** Overview of measurements per visit

**Measurement**	**Baseline visit**	**Follow-up visit 1 (day 50)**	**Follow-up visit 2 (day 197)**	**Follow-up visit 3 (day 379)**
Demographic characteristics	•			
Smoking:				
Tobacco use and quit attempts [41]	•	•	•	•
Fagerström Test for Nicotine Dependence (FTND) [42]	•			
Health perception: (self-constructed questions)				
health concerns	•	•	•	•
risk perception	•	•	•	•
self-exempting beliefs	•	•	•	•
Respiratory health complaints:				
COPD diagnostic questionnaire [43, 44]	•			
Medical Research Council (MRC) dyspnoea scale [45, 46]	•		•	•
Clinical COPD Questionnaire (CCQ) [47, 48]	•	•	•	•
Health-related quality of life:				
EuroQol (EQ-5D) [39, 40]	•	•	•	•
Short-form 36-item questionnaire (SF-36) [49, 50]	•	•	•	•
Chronic Respiratory Questionnaire self-reported (CRQ-SR) [51, 52]	•	•	•	•
Mental health:				
Beck Depression Inventory (BDI) [53]	•	•	•	•
Hospital Anxiety and Depression Scale (HADS) [54–56]	•	•	•	•
Cost diary: measurement of direct en indirect medical and non-medical costs [38]		•	•	•
Physical measurements:				
Physical height and weight	•	•	•	•
Post-bronchodilator spirometry	•			•
Urine cotinine (only in self-reported quitters)		•	•	•

Urine is collected from every self-reported non-smoker during each follow-up visit to validate non-smoking. The urine is kept in a 100 mL plastic cup with a screw cap and temporarily stored in a refrigerator for a maximum of seven days before delivery to the laboratory of the Department of Health Risk Analysis and Toxicology (GRAT) of Maastricht University. The concentration of cotinine in urine is measured by a highly specific radioimmunoassay using monoclonal antibodies [36]. The reagents for the assay are obtained from the Department of Biochemistry, Brandeis University, Massachusetts, USA.

The analysts assessing the urine cotinine levels and all assistants entering data from questionnaires are kept blind to the group allocation of participants.

### Data analysis

The primary outcome measure is prolonged abstinence from smoking during a period of 12 months after the TQD. Prolonged abstinence is defined as follows: abstinent from smoking at all three follow-up visits; at day 50 (approximately 5 weeks after the TQD), day 197 (after six months), and day 379 (after 12 months). Participants are allowed to miss the second follow-up visit (day 197) if they have been abstinent from smoking at the first (day 50) and the last follow-up visit (day 379; interpolation). A participant is defined as abstinent from smoking at a follow-up visit if both of the following two conditions are met:

1. urine cotinine level < 50 ng/mL [37] and

2. self-reported quitter, not having smoked a single cigarette since stopped smoking.

All randomized subjects will be included in the analysis and subjects not showing up at the follow-up visit or with a missing value on one of the two above measures are regarded as smokers (intention-to-treat analysis). Statistical difference in primary outcome will be analyses using Chi-square tests.

The secondary research questions will be analysed as follows (see the last paragraph of the introduction for an overview of all secondary research questions).

1. Baseline predictors of outcome will be analysed by regressing the primary outcome measure (12-month prolonged abstinence) on candidate predictors measured at baseline (such as age, sex, airflow limitation, previous quit attempts, or nicotine dependence) in a multivariate logistic regression model, controlling for treatment group.

2. The health effects of smoking cessation will be analysed by regressing measures of lung function, perceived specific health-related complaints, quality of life, and mental health on abstinence at each follow-up visit using linear regression models.

3. The cost-effectiveness and cost-utility of early detection of airflow limitation in combination with smoking cessation will be analysed using data from the cost diaries [38]. Participants fill out these diaries during three periods of six weeks each: during the intervention until the first follow-up visit (day 50), during the period until the second follow-up visit (day 197), and during the period until the last follow-up visit (day 379). The economic evaluation is based on direct medical costs (e.g. treatment costs, spirometry), direct non-medical costs (e.g. reimbursement of travelling expenses), and on indirect costs (e.g. sickness absence), which are related to respiratory complaints. Effects are measured in physical units (such as number of successful quit attempts, FEV_1_) and Quality Adjusted Life Years (QALY'S) are measured with the EuroQol (EQ-5D) [39,40].

4. The effects of labelling of disease (COPD) will be analysed by mediation and moderation analyses using linear and logistic regression models. The outcome variable in these analyses is abstinence from smoking at the first follow-up visit. Only data from the experimental group and control group 1 will be used.

5. The ethical considerations of early detection of airflow limitation will be analysed in a qualitative analysis using data from the ethical exit interviews which are performed in participants attending the last follow-up visit.

### Ethical approval, review, and registration of the trial

Participants of this study are not fully informed about the real purpose of the study at the beginning, which is to detect and confront smokers with airflow limitation. Participants from control group 1 and control group 2 are not informed about their results of spirometry during the intervention and follow-up period. This approach is necessary to assess the additional effect of early detection of and confrontation with airflow limitation above the effects of individual counselling and medication use. As already explained in the paragraph "informed consent", all participants as well as their GPs will be fully informed about the purpose of the study and the results of spirometry after the last follow-up visit.

This procedure is approved by the medical ethics committee of Maastricht University and Maastricht University Hospital. We believe that it is ethical to withhold information about the results from spirometry to participants of this study for at least two reasons. The first reason is that the smokers participating in this trial would probably not have been diagnosed with airflow limitation outside the trial setting early due to the problem of underdiagnosis of COPD in primary care. The second reason is that all smokers from this trial receive the most effective therapy for airflow limitation from either the RN or their own GP, which is smoking cessation treatment.

Data from the ethical analysis, which is based on the interviews with participants during the last follow-up visit, should provide information about the participants' view on the ethical aspects of this trial.

The protocol of this study was extensively reviewed by the funding organizations; the Dutch Asthma Foundation, PICASSO for COPD, and Maastricht University Hospital. The study is registered at the Netherlands Trial Register (ISRCTN 64481813).

### Time frame

The recruitment, inclusion, randomization, and treatment of participants will start in 2005 and will continue until 2007. The follow-up of participants is planned until the year 2008. All data will be continuously collected, entered, and cleaned. The analysis of data regarding the primary research question will not be initiated before the completion of follow-up and data collection in the last participants (during the course of the year 2008).

## Discussion

We presented the protocol of a study assessing the efficacy of confrontational counselling for smoking cessation in current smokers with not earlier diagnosed mild to moderate airflow limitation (i.e. GOLD stage 1 and 2 COPD). The design of this study is a randomized controlled trial comparing confrontational counselling delivered by a respiratory nurse (RN) combined with nortriptyline for smoking cessation (experimental group), health education and promotion delivered by a RN combined with nortriptyline for smoking cessation (control group 1), and "care as usual" delivered by the GP (control group 2). We hypothesise that early detection of COPD and confrontation with spirometry for smoking cessation is more effective than regular health education and promotion and primary care as usual for smoking cessation.

In the design of this randomized controlled trial, the baseline risk of all participants is the same; they all have previously undetected airflow limitation. Only participants from the experimental group are confronted with their disease. Participants from the two control groups are not informed before the end of the trial. All other factors which are known to be associated with abstinence from smoking are standardised in both the experimental group and control group 1: type of counsellor (RN), type of counselling (face-to-face and by telephone), number and duration of counselling sessions, and type (nortriptyline) and dosage of smoking cessation medication. Therefore, we are able to assess the "net" effect of confronting and counselling smokers with COPD.

There are several critical success factors to be mentioned. A large number of smokers will have to be screened in order to obtain enough eligible participants. No smokers with known airflow limitation are allowed to enter the trial. Also, during the whole recruitment period, candidate participants must not be informed about the real purpose of the study at the beginning; to detect and confront smokers with airflow limitation. This means that a large number of smokers generally interested in quitting must be recruited in order to filter out eligible smokers with airflow limitation.

We expect eligible smokers to have a strong preference to be placed in one of the groups receiving counselling by a RN in combination with smoking cessation medication, because they may find this intervention more effective than care as usual by their own GP. Therefore, we expect a lower compliance in participants from control group 2 and differential lost to follow-up in this group. It should be noted here that the intervention in control group 2 is probably not care as usual for smoking cessation as it appears in primary care. This is because smokers are more or less "referred" from the study team to their GP and are likely to ask for the same smoking cessation medication as participants randomized to the other two groups (nortriptyline).

Blinding of neither participants nor RNs is possible, because we want to assess behavioural interventions. All participants know what kind of counselling they receive, as do the respiratory nurses who provide the counselling. However, analysts assessing the cotinine levels and assistants entering data from questionnaires are blinded for the group allocation of participants.

The primary outcome, 12-month prolonged abstinence from smoking, is estimated from abstinence measures at three time points. This outcome is therefore no perfect measure of continuous outcome, but it is a feasible estimation, which is very usual in this field of research. Abstinence from smoking is the primary endpoint of this study, but can be regarded as a surrogate for the expected long-term positive effects of smoking cessation on health.

## Conclusion

Early intervention in COPD is of paramount importance because of the irreversible and progressive nature of the disease. The use of spirometry for early detection of COPD still is an issue of debate because of a lack of convincing evidence that spirometry has an added positive effect on smoking cessation. A significant and relevant effect of confrontational counselling compared to regular health education and promotion for smoking cessation would provide an argument in favour of early detection of current smokers with airflow limitation. Successful treatment of tobacco dependence in respiratory patients requires repeated intensive interventions. The results of this study may show that RNs are able to deliver this treatment on the and that intensive smoking cessation counselling is more feasible for RNs than for physicians who often lack time.

## Abbreviations

AL = airflow limitation

BMI = Body Mass Index (body weight in kilogram/physical height in meters^2^)

COPD = chronic obstructive pulmonary disease

FC = face-to-face contact session

FEV_1 _= Forced Expiratory Volume in one second

FVC = Forced Vital Capacity

GP = general practitioner

Pack year = (number of cigarettes smoked per day) × (number of years smoking)/20

Post-bd. = post-bronchodilator

RA = research assistant

RN = respiratory nurse

TC = telephone contact session

TQD = target quit date

%pred. = percentage of predicted lung function value

## Competing interests

The author(s) declare that they have no competing interests.

## Authors' contributions

DK is the principal investigator of the study and wrote this manuscript. CP and GW are supervisors and grant applicators. Both contributed to the study methodology and to the writing of the manuscript. MH contributed to the design of the experimental intervention and to the writing of the manuscript. All authors read and approved the final manuscript.

## Pre-publication history

The pre-publication history for this paper can be accessed here:


